# Observational Study Comparing Efficacy and Safety between Neoadjuvant Concurrent Chemoradiotherapy and Chemotherapy for Patients with Unresectable Locally Advanced or Metastatic Gastric Cancer

**DOI:** 10.1155/2020/6931317

**Published:** 2020-09-07

**Authors:** Yung-Sung Yeh, Ming-Yii Huang, Cheng-Jen Ma, Ching-Wen Huang, Hsiang-Lin Tsai, Yen-Cheng Chen, Ching-Chun Li, Fang-Jung Yu, Hsiang-Yao Shih, Jaw-Yuan Wang

**Affiliations:** ^1^Division of Colorectal Surgery, Department of Surgery, Kaohsiung Medical University Hospital, Kaohsiung Medical University, Kaohsiung, Taiwan; ^2^Division of Trauma and Surgical Critical Care, Department of Surgery, Kaohsiung Medical University Hospital, Kaohsiung Medical University, Kaohsiung, Taiwan; ^3^Department of Radiation Oncology, Kaohsiung Medical University Hospital, Kaohsiung Medical University, Kaohsiung, Taiwan; ^4^Department of Radiation Oncology, Faculty of Medicine, College of Medicine, Kaohsiung Medical University Hospital, Kaohsiung Medical University, Kaohsiung, Taiwan; ^5^Division of General and Digestive Surgery, Department of Surgery, Kaohsiung Medical University Hospital, Kaohsiung Medical University, Kaohsiung, Taiwan; ^6^Department of Surgery, Faculty of Medicine, College of Medicine, Kaohsiung Medical University, Kaohsiung, Taiwan; ^7^Division of Gastroenterology, Department of Internal Medicine, Kaohsiung Medical University Hospital, Kaohsiung, Taiwan; ^8^Department of Medicine, College of Medicine, Kaohsiung Medical University, Kaohsiung, Taiwan; ^9^Graduate Institute of Clinical Medicine, College of Medicine, Kaohsiung Medical University, Kaohsiung, Taiwan; ^10^Graduate Institute of Medicine, College of Medicine, Kaohsiung Medical University, Kaohsiung, Taiwan; ^11^Clinical Pharmacogenomics and Pharmacoproteinomics, College of Pharmacy, Taipei Medical University, Taipei, Taiwan; ^12^Drug Development and Value Creation Research Center, Kaohsiung Medical University, Kaohsiung, Taiwan

## Abstract

**Objective:**

Dismal outcomes in patients with locally advanced or metastatic gastric cancer (GC) highlight the need for effective systemic neoadjuvant treatment strategies to improve clinical results. Neoadjuvant multimodality strategies vary widely. This study compared the efficacy, safety, and clinical outcomes of neoadjuvant CCRT and chemotherapy for such patients.

**Materials and Methods:**

Sixty-five patients with histologically confirmed locally advanced or metastatic GC following neoadjuvant CCRT or computed tomography (CT) were retrospectively enrolled between January 2010 and April 2019. Clinical outcomes included response, progression-free survival (PFS), and overall survival (OS), and toxicity was compared between the two groups.

**Results:**

Of the 65 patients, 18 (27.7%) were in the response group (2 patients with a complete response and 16 with a partial response) and 47 (72.3%) in the nonresponse group (29 patients with a stable disease and 18 with a progressive disease). Multivariate analysis revealed no independent response predictor between CCRT and chemotherapy groups (all *P* > 0.05). Furthermore, results revealed no statistical differences in toxicity between the two groups (all *P* > 0.05). With a follow-up median of 12 months (ranging 6–48 months), 12-month OS and PFS were 39.7% and 20.4% in the CCRT group and 30.3% and 13.2% in the chemotherapy group, respectively. The median OS and PFS were 14.0 months (95% CI 9.661–18.339) and 9.0 months (95% CI 6.805–11.195) in the CCRT group and 10.0 months (95% CI 6.523–13.477) and 8.0 months (95% CI 6.927–9.073) in the chemotherapy group, respectively. Both OS (*P*=0.011) and PFS (*P*=0.008) in patients with CCRT were significantly better than those in patients with chemotherapy alone.

**Conclusion:**

Neoadjuvant CCRT achieved more favorable OS and PFS than did neoadjuvant chemotherapy alone, without significant increases of toxicity in patients. However, prospective randomized trials comparing treatment modalities are necessary to confirm the potential advantages of neoadjuvant CCRT.

## 1. Introduction

Gastric cancer (GC) is one of the most common malignancies and, despite a steady decline, GC remains the leading cause of death with widely varying incidence worldwide [1]. Despite earlier detection of GC and considerable advancements in treatments that improve opportunities for survival, its mortality and morbidity rates remain high, with local advanced or distant metastases occurring in up to 60% of patients [2, 3]. In patients with unresectable locally advanced or metastatic GC, the median survival time without chemotherapy is approximately 3-4 months. Complete surgical resection remains the only chance for a cure, and multimodality treatment approaches are implemented to improve survival chances [1]. Thus, dismal outcomes in patients with locally advanced or metastatic GC highlight the need for effective systemic neoadjuvant treatment to improve clinical results.

Recently, several clinical trials have shown that neoadjuvant CCRT can benefit patient survival after surgery for GC [4, 5]. Moreover, neoadjuvant CCRT has more theoretical benefits than neoadjuvant chemotherapy in patients with unresectable locally advanced or metastatic GC, including more favorable progression-free survival (PFS) and overall survival (OS) without a significant toxicity increase in patients [6]. These strategies improve disease-related outcomes more than surgery alone but are associated with higher rates of treatment-related morbidity. Illustrating this fact, only 64% of patients in the Intergroup-0116 trial and 42% in the Medical Research Council Adjuvant Gastric Infusional Chemotherapy trial could complete their prescribed treatment courses [7, 8]. Hence, the establishment of a more precise treatment protocol that appropriately selects patients and provides specific therapy is ongoing.

Patients with more locally advanced GC who underwent gastrectomy after preoperative CCRT experienced outcomes that varied between favorable and poor. In this study, we presented our experience with neoadjuvant CCRT versus chemotherapy alone in patients with unresectable locally advanced or metastatic GC and compared baseline characteristics, efficacy, and safety between the two groups.

## 2. Materials and Methods

### 2.1. Data Source and Study Design

This study was conducted at Kaohsiung Medical University Hospital with approval from the hospital's institutional review board (KMUHIRB-20130022), and informed consent was obtained from all patients. The study started in January 2010, and as of April 2019, 65 patients with histologically confirmed locally advanced T4 or metastatic GC have been included. In the current study, diagnostic laparoscopy is not routinely performed in metastatic GC patients with dissemination, but diagnostic laparoscopy will be performed where image studies cannot confirm if the curative-intent resection could be performed in locally advanced T4 GC patients without distant metastasis. Baseline investigations consist of blood tests, gastroendoscopy with tumor biopsy samples, complete history review and physical examination, and image studies (i.e., chest radiography, abdominal computed tomography (CT), and additional magnetic resonance imaging (MRI) if the CT scan could not clarify the cancer stage). TNM classification was determined according to the American Joint Commission on Cancer/Union for International Cancer Control criteria [9].

### 2.2. Patient Selection

Patients with histologically proven locally advanced T4 or metastatic GC are eligible for this study. Patients should be at least 18 years old with an Eastern Cooperative Oncology Group performance status of 0–2. Patients must have adequate hematological, renal, and liver function. Exclusion criteria include central nervous system metastases or previous malignancy, active infections, or serious concurrent medical illness (i.e., clinically significant cardiac disease or liver disease, known peripheral neuropathy), life expectancy <3 months, prior radiotherapy or chemotherapy, and inability to receive neoadjuvant therapy. Considering the eligibility criteria of the patient, neoadjuvant chemoradiotherapy or chemotherapy was chosen after shared decision-making with patients and family meetings according to the real-world situation in Taiwan.

We included patients with distant metastasis such as liver, lung, and bone metastasis in this analysis. Curative surgery was not suitably applied to GC patients with liver, lung, and bone metastasis with neoadjuvant CCRT or chemotherapy. GC patients with liver, lung, or bone metastasis would undergo an operation if good response following neoadjuvant CCRT or chemotherapy is obtained; thereafter, conversion to surgery will be performed.

### 2.3. Clinicopathological Characteristics

Clinicopathological characteristics, such as age, sex, tumor size, tumor invasion depth, lymph node metastasis, clinical TNM status, vascular invasion, perineural invasion, tumor location, histological tumor differentiation grade, pretreatment metastasis site, and pretreatment serum carcinoembryonic antigen (CEA) level, were analyzed. Her-2 expression was not often tested as trastuzumab was not reimbursed in Taiwan. Additionally, positive Her-2 expression was approximately only 6% in Taiwan [10]; therefore, Her-2 expression was not routinely examined for neoadjuvant setting in the current study. This study aimed to explore the efficacy and safety profile of preoperative CCRT in locally advanced or metastatic GC versus preoperative chemotherapy.

### 2.4. Treatment Modalities

Currently, no particular neoadjuvant protocol is internationally regarded as superior in the multimodal therapeutic armamentarium. Interpretation of trial results is controversial, which results in strong interinstitutional differences concerning radiotherapy and chemotherapy sequence for the treatment of patients with locally advanced T4 or metastatic GC. Whether preoperative chemotherapy or CCRT should be recommended for the treatment of patients with locally advanced T4 or metastatic GC remains uncertain. Furthermore, both options are suggested by guidelines supported by the National Comprehensive Cancer Network. In this study, we compared the survival data of patients with locally advanced T4 or metastatic GC, treated with either CCRT or chemotherapy, based on real-world data from one institution, and we additionally reviewed the current literature.

### 2.5. Chemotherapy

The 65 patients were treated with an mFOLFOX-4-based regimen that comprised the following. On day 1, oxaliplatin (85 mg/m^2^) and leucovorin (200 mg/m^2^) were administered over a 2-hour period, followed by a 48-hour continuous infusion of 5-FU at a dose of 2400 mg/m^2^ every 2 weeks. The primary endpoints of this study were response rate, PFS, and OS. The secondary endpoints were acute toxicities during preoperative CCRT or chemotherapy.

### 2.6. Radiotherapy

Three-dimensional conventional radiotherapy (3D-CRT) was delivered using a 2100 C/D linear accelerator (Varian Medical Systems, Palo Alto, CA, USA). For the 3D-CRT plan, we used anterior–posterior and posterior–anterior fields with photon energy at 10 MV. The dose specification for 3D-CRT encompassed the planning target volume (PTV) in all directions within the 95% isodose line. Volumes receiving more than 110% of the dose prescribed to the PTV were minimized. Reference points were selected either in the central part of PTV or at the intersection of beam axes from the International Commission on Radiation Units and Measurements (ICRU; Reports 50 and 62). The radiation portal fields were designed as follows: (i) proximal one-third/cardia/esophagogastric junction primaries included 3–5 cm of the distal esophagus, medial left hemidiaphragm, adjacent pancreatic body, and nodal areas at risk including adjacent paraesophageal, perigastric, suprapancreatic, and celiac lymph nodes; (ii) middle one-third/body primaries included pancreatic body and nodal areas at risk including perigastric, suprapancreatic, celiac, splenic hilar, porta hepatic, and pancreaticoduodenal lymph nodes; and (iii) distal one-third/antrum/pylorus primaries included pancreatic head, 3–5 cm margin duodenal stump margin if gross lesions extended to the gastroduodenal junction, and nodal areas at risk including perigastric, suprapancreatic, celiac, splenic hilar, porta hepatic, and pancreaticoduodenal lymph nodes. Radiotherapy consisted of 45–50.4 Gy in 25–28 fractions over 5 weeks.

Image-guided (IG) intensity-modulated radiotherapy (IMRT) plans were generated either with a Hi-Art helical tomotherapy unit, version 2.2.4.1 (TomoTherapy, Inc., Madison, WI, USA), or Eclipse, version 8.6 (Varian Medical Systems). The tomotherapy unit combined rotational IMRT with translational movement from the couch. A fixed-jaw mode with a field width of 2.5 or 5 cm was used for treatment planning. The pitch varied from 0.215 to 0.287. The modulation factor ranged from 2 to 3 depending on homogeneity and conformity. The gross tumor volume encompassed gastric tumors and clustered lymph nodes or lymph nodes with diameters greater than 1 cm. The clinical target volume (CTV) included the primary tumor, and adjacent lymphatic drainage depended on primary tumor location. Superior, inferior, and radial margins of 5–7 mm outside the CTV were added to form the PTV.

In the IG-IMRT group, the tumor and boost beams were combined into one integrated treatment plan; thus, these patients were treated with the same plan for each fraction throughout the entire course of radiotherapy. Fractionation schemes comprised 25 daily fractions of 1.8 Gy to the pelvis and 2 Gy to the gastric tumor and involved nodes. Optimization reduced doses for the bowel, kidney, liver, and spinal cord. These constraints were also applied to IMRT treatment plans on Varian and comprised beams with multileaf collimator shielding conforming to the PTV. The goal was to encompass the PTV in all directions within the 95% isodose line. Volumes receiving more than 110% of the dose prescribed to the PTV were minimized. Volumetric arc therapy was used when suitable. IMRT plans were reviewed using ICRU 83 recommendations. Before each RT fraction, patients were repositioned according to image guidance through megavoltage or cone-beam CT, which was coregistered with a planning kilovoltage CT. A dose of 50 Gy was administered to the PTV50 (tumor and enlarged nodes) and 45 Gy to the PTV45 (adjacent high-risk nodal area) through a simultaneous integrated boost scheme in the IG-IMRT group. All dose schedules were administered 5 days per week.

### 2.7. Toxicity

Safety and toxicity were evaluated in each cycle by using the National Cancer Institute Common Terminology Criteria for Adverse Events (CTCAE), version 4.03 (https://ctep.cancer.gov/protocoldevelopment/electronic_applications/ctc.htm, accessed in 2018). Peripheral neuropathy was graded according to the following oxaliplatin-specific scale: grade 1, paresthesia or dysesthesia of short duration with complete recovery before the next cycle; grade 2, paresthesia persisting between two cycles without functional impairment; and grade 3, permanent paresthesia interfering with function [2, 3]. Neoadjuvant chemotherapy was discontinued in cases of unacceptable toxicity (>grade 3), disease progression, or patient refusal to continue treatment [2, 3].

### 2.8. Evaluation of Response and Efficacy Assessment

Physical examination, liver and kidney function tests, complete blood count and serum CEA level examination, and electrocardiogram were performed before and after every two weeks of treatment. Abdominal CT and additional MRI are performed every 3 months during chemotherapy; if necessary, a chest X-ray is performed annually. A bone scan or positron emission tomography scan is selectively performed to display images of suspicious findings at specific locations of CT or MRI and suspicious metastases. All enrolled patients were followed up every 3 months until the last visit or death. The median follow-up time for all patients was 12 months (range, 6–48 months).

Patient responses were classified according to the Response Evaluation Criteria in Solid Tumors [2, 3]. Complete remission (CR) is the disappearance of treatment for all target cancer lesions. Partial response (PR) is a reduction of at least 30% of the sum of the longest diameters of metastatic lesions, with no signs of new lesions. Progressive disease (PD) is a cumulative increase in the longest diameter of the target lesion by at least 20%, and the smallest sum of the longest diameters recorded before the patient begins treatment is used as a reference. The PD can also recognize one or more new lesions. The contraction rate of stable disease (SD) is not sufficient to meet the PR criteria, and the increase is not sufficient to meet the PD criteria [2]. Finally, PFS was determined by measuring the time interval between the start of neoadjuvant CCRT or chemotherapy and the first record of progression, regardless of the patient's treatment status or final follow-up, while OS was measured by neoadjuvant CCRT or chemotherapy to chemotherapy. The starting time interval is determined. Date of death or last follow-up [2, 3].

### 2.9. Statistical Analysis

Continuous variables are presented as the mean ± SD, and dichotomous variables are presented as numbers and percentage values. All data were analyzed using SPSS (version 20.0, SPSS Inc., Chicago, IL, USA). The chi-square test was used to compare toxicity and outcomes. Univariate analyses and a multivariate Cox proportional hazard regression were performed to evaluate independent predictors. PFS and OS were calculated and plotted according to Kaplan–Meier methods, and the log-rank test was used to compare time-to-event distribution. A *P* value of less than 0.05 was considered statistically significant.

## 3. Results

From January 2010 to April 2019, 65 patients with locally advanced or metastatic GC who received first-line neoadjuvant CCRT and chemotherapy were enrolled. The type of preoperative therapy, clinical features, and tumor characteristics are listed in Table 1. All 65 patients received at least six chemotherapy cycles with or without preoperative radiotherapy and were eligible for efficacy and toxicity analysis. Out of the 65 patients, 47 patients (72.3%) were observed to have T4 tumors, 24 patients exhibited distant metastasis (36.9%), and all patients had N+ disease. Otherwise, the patients included 42 men and 22 women who had a mean age of 65.4 years (ranging 31–85 years).

Diagnostic laparoscopy may be useful for the diagnosis of distant metastasis or tumor invasion to the surrounding tissues, such as dissemination or tumor invasion to the adjacent organs before CCRT or chemotherapy. Diagnostic laparoscopy was performed here to confirm whether locally advanced T4 GC patients had dissemination or not before the operation for locally advanced T4 GC patients without distant metastasis (41 patients, 63.1%).

Out of these 65 patients, 25 patients (38.5%) underwent resection with curative intent. According to the pathological reports of these 25 patients, there were 10 patients (15.4%) with positive vascular invasion and 11 patients (16.9%) with perineural invasion. Histologically, 15 tumors (23.1%) were moderately differentiated, and 50 tumors (76.9%) were poorly differentiated, where no tumors were well-differentiated.

In this group of patients, after neoadjuvant therapy, the most common primary tumor sites were antrum (47.7%), followed by cardia (27.7%), body (27.7%), diffuse (27.7%), and gastric stump (4.6%). And the most common sites of distant metastases were peritoneum (12.3%), liver (10.8%), lungs (9.2%), and ovaries (4.6%). Among all 65 patients, there were 17 patients (26.2%) with T downstaging and 16 patients (24.6%) with N downstaging. Additionally, 18 patients (27.7%) had TNM downstaging after neoadjuvant CCRT and chemotherapy.

Surgeons and radiation oncologists recorded acute toxicities according to the CTCAE, version 4.03 (https://ctep.cancer.gov/protocoldevelopment/electronic_applications/ctc.htm, accessed in July 2019). The most frequent toxicities of grade 3/4 hematological and nonhematological toxicities are shown in Table 2. Major grade 3/4 hematological toxicities included neutropenia in four patients (6.2%), febrile neutropenia in three patients (4.6%), and thrombocytopenia in one patient (1.5%). Other grade 3/4 nonhematological toxicities were nausea/vomiting (15.4%), anorexia/fatigue (13.8%), abnormal liver function (7.7%), abnormal renal function (7.7%), peripheral neuropathy (6.2%), stomatitis (6.2%), diarrhea (6.2%), and constipation (4.6%). No additional safety concerns were identified in the current study. No deaths were associated with the study treatment. Among these adverse events, these results revealed no statistical difference in toxicities between the two groups (all *P* > 0.05).

Neoadjuvant CCRT was performed in 30 patients (46.2%) and chemotherapy in 35 patients (53.8%). No significant association was observed between these two neoadjuvant treatment modalities and baseline clinicopathological features, namely age, sex, tumor size, clinical T status, clinical N status, vascular invasion, perineural invasion, clinical TNM stage, pretreatment serum CEA level, and posttreatment serum CEA level (Table 3, all *P* > 0.05). The incidence of postoperative complications may affect OS or PFS. Few patients had postoperative complications in the results (2 patients with small bowel obstruction; 2 patients with surgical wound infection; 3 patients with urinary tract infection; 3 patients with pneumonia, 2 patients with anastomotic insufficiency). Moreover, no significant association was observed between these two neoadjuvant treatment modalities and surgical outcomes, such as operative time, estimated blood loss (Table 3, all *P* > 0.05).

All patients were evaluated for tumor response. Major responses were observed in 18 patients (27.7%), of which 2 patients (3.1%) underwent CR and 16 patients (24.6%) underwent PR. In addition, 29 patients (44.6%) exhibited SD, and 18 patients (27.7%) had PD. Disease control rates were observed in 48 patients (73.9%) in the CCRT group and 25 patients (83.3%) in the chemotherapy group. Among the 65 patients, 18 (27.7%) were categorized into the response group (2 CR and 14 PR) and 47 (72.3%) into the nonresponse group (29 SD and 18 PD). From the univariate analysis of the correlation between the response group and clinicopathological features, we observed no significant differences between preoperative CCRT and chemotherapy groups (Table 4). In Table 4, there are 10 response patients in the CCRT group and 8 response patients in the chemotherapy group. The mean age of the patients is 62 years old (range 38–80 years old) in the CCRT group and 66 years old (range 45–82 years old) in the chemotherapy group. No significant differences in age, sex, tumor size, clinical T status, clinical N status, vascular invasion, perineural invasion, clinical TNM stage, pretreatment serum CEA level, and posttreatment serum CEA level were observed (all *P* > 0.05). In addition, the multivariate logistic regression analysis revealed no independent predictor of response between CCRT and chemotherapy groups (all *P* > 0.05).

**Figure 1:**
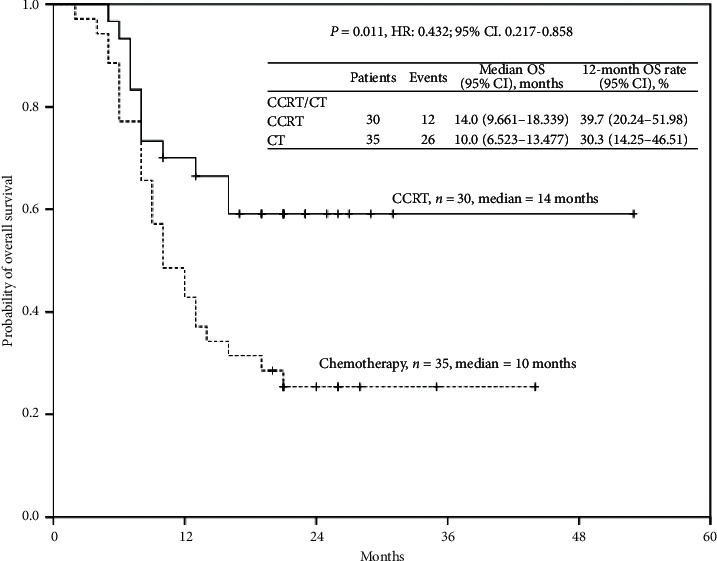
Cumulative overall survival rates of the 65 enrolled patients with unresectable locally advanced or metastatic gastric cancer undergoing neoadjuvant concurrent chemoradiotherapy or chemotherapy, as assessed using the Kaplan–Meier method. The differences in survival rates were analyzed using the log-rank test.

**Figure 2:**
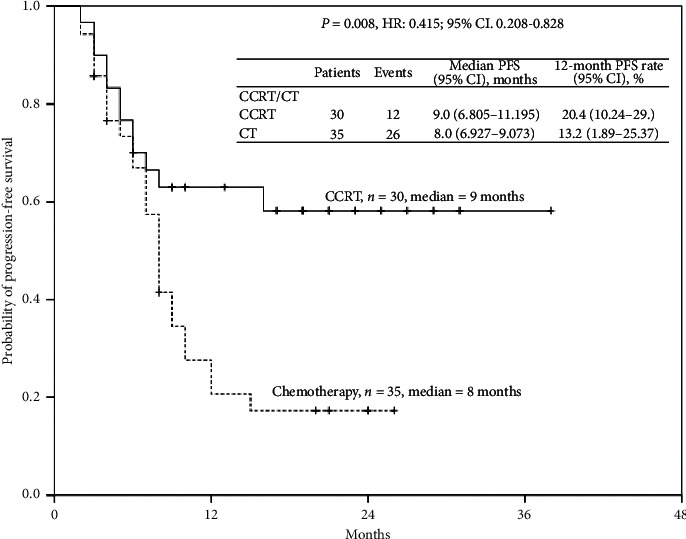
Cumulative progression-free survival rates of the 65 enrolled patients with unresectable locally advanced or metastatic gastric cancer undergoing neoadjuvant concurrent chemoradiotherapy or chemotherapy, as assessed using the Kaplan–Meier method. The differences in survival rates were analyzed using the log-rank test.

PFS and OS based on neoadjuvant CCRT or chemotherapy are displayed in Figures 1 and 2. Median OS and PFS were 14.0 months (95% CI: 9.661–18.339) and 9.0 months (95% CI: 6.805–11.195), respectively, in the CCRT group patients. By contrast, median OS and PFS were 10.0 months (95% CI: 6.523–13.477) and 8.0 months (95% CI: 6.927–9.073), respectively, in chemotherapy group patients. The 12-month OS rates in patients with CCRT or chemotherapy were 39.7% and 30.3%, respectively, whereas the 12-month PFS rates in patients with CCRT or chemoradiotherapy were 20.4% and 13.2%, respectively.

Notably, both OS and PFS in patients with CCRT were better than those in patients with chemotherapy (*P*=0.011 and *P*=0.008, respectively). CCRT, rather than chemotherapy, appeared effective for achieving better survival benefit.

## 4. Discussion

In our previous study, GC is still one of the leading malignant tumors in the world. In the past few decades, the treatment of patients with locally advanced or metastatic GC has not changed substantially [2]. A neoadjuvant strategy may increase the likelihood of completing multimodality therapy, particularly when surgical management is associated with significant morbidity and complications that may preclude timely adjuvant therapy [9–15]. Preoperative concurrent chemoradiotherapy (CCRT) is a well-established primary treatment modality in other gastrointestinal malignancies including esophageal [9, 11] and rectal cancer [12, 13]. This treatment approach involves sterilizing the surgical field, potentially reducing the risk of local tumor dissemination at resection. Preoperative CCRT may also allow smaller and more accurate radiation treatment fields, which could improve treatment tolerance and chemotherapy effects [14]. The application of neoadjuvant radiotherapy for patients with unresectable locally advanced or metastatic GC has several additional and distinct advantages. The presence of intact tumors and preserved normal anatomy facilitates treatment planning and may limit toxicity to adjacent organs. By contrast, adjuvant radiotherapy mandates high doses and large treatment fields that may increase toxicity.

In recent years, a multidisciplinary treatment approach including preoperative chemotherapy, radiotherapy, and target therapy has emerged for advanced or metastatic GC, resulting in increased curability and improved survival [14]. Recent studies have suggested that neoadjuvant chemotherapy in patients with locally advanced or metastatic GC can enable curative resection and improve survival [15, 16]. In our previous study, we observed that patients with locally advanced or metastatic GC who received neoadjuvant chemotherapy had better survival and quality of life than patients who received only supportive care [2, 3].

Low resectability is the main cause of poor prognosis in patients with locally advanced or metastatic GC who cannot undergo curative surgery. Patients who underwent curative surgery experienced better outcomes than did those who did not undergo surgery [2, 17]. However, radical surgery in patients with locally advanced or metastatic GC is limited because of the high risk of perioperative and postoperative morbidity and mortality and the low rate of resection [18]. Timely implementation of the radical resection of cancer is a key step toward favorable therapeutic results. Therefore, improving survival rates to increase resection rates for the treatment of advanced GC is necessary.

A neoadjuvant approach can be applied broadly; however, its advantages may be most pronounced in these specific patient sunsets. In previous prospective studies, patients treated with neoadjuvant CCRT have exhibited higher response rates than did those with chemoradiotherapy alone. Numerous clinical trials have shown that neoadjuvant CCRT is feasible, and resection rates are higher in patients treated with CCRT [19, 20]. Many findings are expected from trials that explore ways of improving preoperative treatment strategies for locally advanced or metastatic GC [21–25]. Another main reason for the addition of radiotherapy to preoperative chemotherapy is to achieve better local control. Radiotherapy may reduce focal inflammatory edema and fibrous adhesion of the tumor after chemotherapy. A previous phase III clinical trial reported that neoadjuvant CCRT significantly reduced locoregional recurrence from 34% to 14%, with only 1% in-field recurrence [20, 21]. In a Japanese pilot study, no local recurrence was observed after neoadjuvant CCRT [22].

The present study demonstrated favorable OS and PFS responses for patients with locally advanced or metastatic GC treated with CCRT. In our study, 18 (27.7%) of the 65 patients were categorized into the response group and the remaining 47 patients (72.3%) into the nonresponse group. The results of this study indicated that patients who had locally advanced or metastatic GC with the treatment of neoadjuvant CCRT tended to have better PFS and OS than did patients with neoadjuvant chemotherapy alone. We did not observe any difference in the morbidity rate between the two treatments with tolerable toxicity and safety. Our study has several limitations. First, this was a relatively small study of a limited sample size. Neoadjuvant CCRT achieved a more favorable OS and PFS than did neoadjuvant chemotherapy alone, but no significant increases of toxicity, response rate, disease control rate, and resectability were noted.

Therefore, these results supported the use of neoadjuvant CCRT in the treatment of patients with locally advanced or metastatic GC. However, additional studies conducted with larger sample sizes and careful patient monitoring are required to confirm these findings. More specially designed studies and reliable biological indicators of real functional status are needed to properly select patients for multimodal treatment. The results of such studies could be used to significantly demonstrate therapeutic efficacy in the treatment of patients with locally advanced or metastatic GC.

## 5. Conclusion

Neoadjuvant CCRT achieved a more favorable OS and PFS than did neoadjuvant chemotherapy alone, without significant increases of toxicity in patients with locally advanced or metastatic GC. A prospective randomized trial comparing both treatment modalities was conducted to demonstrate the efficacy of neoadjuvant CCRT.

## Figures and Tables

**Table 1:** Baseline characteristics of 65 locally advanced/metastatic gastric cancer patients.

		*N* = 65 (%)
Age, years		
Mean (range)		65.4 (31–85)
≧65 years		33 (50.8)
<65 years		32 (49.2)
Gender		
Male		42 (64.6)
Female		23 (35.4)
Tumor size		
<5 cm		36 (55.4)
≧5 cm		29 (44.6)
Clinical T status		
T4		47 (72.3)
T3		18 (27.7)
Clinical *N* status		
*N*1		10 (15.4)
*N*2 + *N*3		55 (84.6)
Vascular invasion		
Positive		10 (15.4)
Negative		13 (20.0)
ND		42 (64.6)
Perineural invasion		
Positive		11 (16.9)
Negative		12 (18.5)
ND		42 (64.6)
Clinical TNM stage		
Stage III		41 (63.1)
Stage IV		24 (36.9)
Tumor location		
Cardiac		18 (27.7)
Body		18 (27.7)
Antrum		31 (47.7)
Diffuse (borrmann IV)		18 (27.7)
Stump		3 (4.6)
Histology		
Well-differentiated	0	
Moderately differentiated		15 (23.1)
Poorly differentiated		50 (76.9)
Metastasis site		
Peritoneum carcinomatosis		8 (12.3)
Liver		7 (10.8)
Lung		6 (9.2)
Bone		2 (3.1)
Ovary		3 (4.6)
Bladder		1 (1.5)
Pretreatment CEA (ng/ml)		
≧5		16 (24.6)
<5		49 (75.4)
Posttreatment CEA (ng/ml)		
≧5		20 (30.8)
<5		45 (69.2)
TNM downstaging		
Yes		18 (27.7)
No		47 (72.3)
T downstaging		
Yes		17 (26.2)
No		48 (73.8)
N downstaging		
Yes		16 (24.6)
No		49 (75.4)
R0/R1 resection		
Yes		25 (38.5)
No		40 (61.5)
CCRT or chemotherapy		
CCRT		30 (46.2)
Chemotherapy		35 (53.8)

ND=Not done (38 patients: unresectable tumor; 2 patients: complete response after neoadjuvant chemoradiotherapy); CEA: carcinoembryonic antigen; CCRT = concurrent chemoradiotherapy.

**Table 2:** Grade 3/4 toxicities according to the National Cancer Institute Common Terminology Criteria for Adverse Events.

	Total *N* = 65 (%)	CCRT *N* = 30 (%)	Chemotherapy *N* = 35 (%)	*P* value
*Hematologic*	8 (12.3)	3 (10.0)	5 (14.3)	1.000
Neutropenia	4 (6.2)	2 (6.7)	2 (5.7)	1.000
Febrile neutropenia	3 (4.6)	1 (3.3)	2 (5.7)	1.000
Thrombocytopenia	1 (1.5)	0	1 (2.9)	1.000
*Nonhematologic*	44 (67.7)	20 (66.7)	24 (68.6)	0.768
Nausea/vomiting	10 (15.4)	5 (16.7)	5 (14.3)	1.000
Anorexia/fatigue	9 (13.8)	4 (13.3)	5 (14.3)	0.708
Abnormal liver function	5 (7.7)	2 (6.7)	3 (8.6)	1.000
Abnormal renal function	5 (7.7)	2 (6.7)	3 (8.6)	1.000
Peripheral neuropathy	4 (6.2)	2 (6.7)	2 (5.7)	1.000
Stomatitis	4 (6.2)	3 (10.0)	1 (2.9)	0.270
Diarrhea	4 (6.2)	2 (6.7)	2 (5.7)	1.000
Constipation	3 (4.6)	0	3 (8.6)	0.255

CCRT = concurrent chemoradiotherapy.

**Table 3:** Correlation between CCRT or chemotherapy and clinicopathologic features in 65 locally advanced/metastatic gastric cancer patients.

	Total *N* = 65	CCRT *N* = 30 (%)	Chemotherapy *N* = 35 (%)	*P* value
Age, years				
≧65 years	33 (50.8)	10 (33.3)	23 (65.7)	0.013
<65 years	32 (49.2)	20 (66.7)	12 (34.3)	
Gender				
Male	42 (64.6)	22 (73.3)	20 (57.1)	0.202
Female	23 (35.4)	8 (36.7)	15 (42.9)	
Tumor size				
<5 cm	36 (55.4)	13 (43.3)	23 (65.7)	0.085
≧5 cm	29 (44.6)	17 (56.7)	12 (34.3)	
Clinical T status				
T4	47 (72.3)	21 (70.0)	26 (74.3)	0.784
T3	18 (27.7)	9 (30.0)	9 (25.7)	
Clinical *N* status				
*N*1	10 (15.4)	5 (16.7)	5 (14.3)	1.000
*N*2 + *N*3	55 (84.6)	25 (83.3)	30 (85.7)	
Vascular invasion				
Positive	10 (43.5)	3 (33.3)	7 (50.0)	0.680
Negative	13 (56.5)	6 (66.7)	7 (50.0)	
Perineural invasion				
Positive	11 (47.8)	4 (44.4)	7 (50.0)	1.000
Negative	12 (52.2)	5 (55.6)	7 (50.0)	
Clinical TNM stage				
Stage III	41 (63.1)	20 (66.7)	21 (60.0)	0.615
Stage IV	24 (36.9)	10 (33.3)	14 (40.0)	
Pretreatment CEA (ng/ml)				
≧5	16 (24.6)	4 (13.3)	12 (34.3)	0.082
<5	49 (75.4)	26 (86.7)	23 (65.7)	
Posttreatment CEA (ng/ml)				
≧5	20 (30.8)	9 (30.0)	11 (31.4)	1.000
<5	45 (69.2)	21 (70.0)	24 (68.6)	
Response rate				
CR + PR	18 (27.7)	10 (33.3)	8 (22.9)	0.411
SD + PD	47 (72.3)	20 (66.7)	27 (77.1)	
Disease control rate				
CR + PR + SD	48 (73.9)	25 (83.3)	23 (65.7)	0.158
PD	17 (26.1)	5 (16.7)	12 (34.2)	
R0/R1 resection				
Yes	25 (35.5)	11 (36.7)	14 (40.0)	0.804
No	40 (61.5)	19 (63.3)	21 (60.0)	
Surgical outcomes (*N* = 25) operating time (median, minute)	—	218	263	0.059
Estimated blood loss (median, ml)	—	202	320	0,142

CCRT: concurrent chemoradiotherapy; CEA: carcinoembryonic antigen; CR = complete response; PR = partial response; SD = stable disease; PD = progressive disease.

**Table 4:** Univariate and multivariate analysis of predictors of response status in 65 locally advanced/metastatic gastric cancer patients.

Variables	Response	Nonresponse	Univariate	Multivariate analysis	*P* value
	(*n* = 18) (%)	(*n* = 47) (%)	*P* value	Odds ratio (95% CI)	
Age, years (<65 years/≧65)	10 (55.6)/8 (44.4)	22 (46.8)/25 (53.2)	0.587	0.704 (0.236–2.098)	0.551
Gender (male/female)	12 (66.7)/6 (33.3)	30 (63.8)/17 (36.2)	1.000	1.133 (0.360–3.567)	0.869
Tumor size (<5/≧5 cm)	10 (55.6)/8 (44.4)	26 (55.3)/21 (44.7)	1.000	0.990 (0.332–2.955)	0.871
Clinical T status (T3/T4)	8 (44.4)/10 (55.6)	10 (21.3)/37 (78.7)	0.073	0.338 (0.106–1.081)	0.101
Clinical N status (*N*1/*N*2 + *N*3)	5 (27.8)/13 (72.2)	5 (10.6)/42 (89.4)	0.124	0.310 (0.077–1.239)	0.202
Vascular invasion (negative/positive/miss)	5 (27.8)/4 (22.2)/9 (50.0)	8 (17.0)/6 (12.8)/33 (70.2)	1.000	1.067 (0.197–5.769)	1.000
Perineural invasion (negative/positive/miss)	5 (27.8)/4 (22.2)/9 (50.0)	7 (14.9)/7 (14.9)/33 (70.2)	1.000	0.800 (0.149–4.297)	1.000
Clinical TNM stage (III/IV)	11 (61.1)/7 (38.9)	30 (63.8)/17 (36.2)	1.000	1.123 (0.367–3.438)	0.779
Pretreatment CEA (<5/≧5) (ng/ml)	14 (77.8)/4 (22.2)	35 (74.5)/12 (25.5)	1.000	0.833 (0.229–3.028)	1.000
Posttreatment CEA (<5/≧5) (ng/ml)	15 (83.3)/3 (16.7)	30 (63.8)/17 (36.2)	0.148	0.353 (0.089–1.396)	0.286
CCRT/Chemotherapy	10 (55.6)/8 (44.4)	20 (42.5)/27 (57.5)	0.411	1.688 (0.565–5.043)	0.511
DCR (yes/no)	18 (100.0)/0	30 (63.8)/17 (36.2)	0.003	1.600 (1.285–1.992)	0.002
R0/R1 resection (yes/no)	11 (61.1)/7 (38.9)	14 (29.8)/33 (70.2)	0.026	3.704 (1.190–11.527)	0.020

CEA: carcinoembryonic antigen; CCRT = concurrent chemoradiotherapy; DCR = disease control rate.

## Data Availability

The datasets supporting the conclusions of this manuscript are included in the article. The raw data are available from the corresponding author upon request.

## Abbreviations

3D-CRT: Three-dimensional conventional radiotherapy
5-FU: 5-fluorouracil
AJCC: American Joint Committee on Cancer
CCRT: Concurrent chemoradiotherapy
CEA: Carcinoembryonic antigen
CR: Complete response
CT: Computed tomography scan
CTV: Clinical target volume
FOLFOX: Chemotherapy of 5-fluorouracil/leucovorin/oxaliplatin
GC: Gastric cancer
ICRU: International Commission on Radiation Units and Measurements
IG: Image-guided
IMRT: Intensity-modulated radiotherapy
MRI: Magnetic resonance image
NCI CTCAE: National Cancer Institute Common Terminology Criteria
OS: Overall survival
PD: Progressive disease
PFS: Progression-free survival
PR: Partial response
PTV: Planning target volume
SD: Stable disease.
